# Characterisation and Analysis of the *Aegilops* *sharonensis* Transcriptome, a Wild Relative of Wheat in the *Sitopsis* Section

**DOI:** 10.1371/journal.pone.0072782

**Published:** 2013-08-08

**Authors:** Costas Bouyioukos, Matthew J. Moscou, Nicolas Champouret, Inmaculada Hernández-Pinzón, Eric R. Ward, Brande B. H. Wulff

**Affiliations:** The Sainsbury Laboratory, Norwich, United Kingdom; Ben-Gurion University, Israel

## Abstract

*Aegilops* *sharonensis* Eig (Sharon goatgrass) is a wild diploid relative of wheat within the *Sitopsis* section of *Aegilops* . This species represents an untapped reservoir of genetic diversity for traits of agronomic importance, especially as a source of novel disease resistance. To gain a foothold in this genetic resource, we sequenced the cDNA from leaf tissue of two geographically distinct *Ae* *. sharonensis* accessions (1644 and 2232) using the 454 Life Sciences platform. We compared the results of two different assembly programs using different parameter sets to generate 13 distinct assemblies in an attempt to maximize representation of the gene space in *de novo* transcriptome assembly. The most sensitive assembly (71,029 contigs; N50 674 nts) retrieved 18,684 unique best reciprocal BLAST hits (BRBH) against six previously characterised grass proteomes while the most specific assembly (30,609 contigs; N50 815 nts) retrieved 15,687 BRBH. We combined these two assemblies into a set of 62,243 non-redundant sequences and identified 139 belonging to plant disease resistance genes of the nucleotide binding leucine-rich repeat class. Based on the non-redundant sequences, we predicted 37,743 single nucleotide polymorphisms (SNP), equivalent to one per 1,142 bp. We estimated the level of heterozygosity as 1.6% in accession 1644 and 30.1% in 2232. The *Ae* *. sharonensis* leaf transcriptome provides a rich source of sequence and SNPs for this wild wheat relative. These sequences can be used with existing monocot genome sequences and EST sequence collections (e.g. barley, *Brachypodium* , wheat, rice, maize and *Sorghum* ) to assist with genetic and physical mapping and candidate gene identification in *Ae* *. sharonensis* . These resources provide an initial framework to further build on and characterise the genetic and genomic structure of *Ae* *. sharonensis* .

## Introduction

Wheat ( *Triticum* spp) is one of the world’s most important cereal crops; it is a major staple across much of the developed and developing world, supplying ~20% of human dietary calories [1]. As the global population continues to increase, wheat yields must be sustainably secured [2]. However, the cultivation of wheat in sub-optimal environments and climate change renders the crop vulnerable to both abiotic stresses and new emerging pests and diseases.

*Aegilops* *sharonensis* Eig (common name: Sharon goatgrass) is a wild relative of wheat belonging to the *Sitopsis* section of *Aegilops* , the genus most closely related to *Triticum* [3]. *Ae* *. sharonensis* contains *Gametocidal* genes that restrict interspecies hybridization [4], which has limited traditional introgression of *Ae* *. sharonensis* genes into wheat, making this species an untapped reservoir of genetic diversity for wheat improvement [5]. Modern breeding techniques and transgenic technology, however, provide the potential to exploit this germplasm. Recently, *Ae* *. sharonensis* has received attention as a source of resistance to the Ug99 race of wheat stem rust [6,7], which poses a major threat to global food security [8]. Several attributes make *Ae* *. sharonensis* attractive for identifying genes for wheat improvement: (i) it is diploid, (ii), wild accessions collected from its range along the Israeli-Lebanon coastline display high genetic diversity [9], and (iii) pure isolines can be readily maintained in the laboratory.

The synteny of the cereal genomes [10] allows existing resources developed in other grasses such as rice, *Brachypodium* , *Sorghum* , maize, barley, and wheat and its direct ancestors [11–21] to be used in genetic and genomic analysis of *Ae* *. sharonensis* . Full genome sequences and high-density genetic maps are available for *Brachypodium* , rice, maize and barley. Barley, separated by ~12 million years of evolution from *Ae* *. sharonensis* and sharing the same base chromosome number (2n=14), is likely to provide better genome-wide synteny than *Brachypodium* , rice or maize, which are separated by approximately 36, 47 and 53 million years of evolution [12,22]. Thus, characterisation of the *Ae* *. sharonensis* gene space represents a first important step to make use of the existing resources in other model grass systems to pursue useful traits in *Ae* *. sharonensis* .

The genome size of *Ae* *. sharonensis* is estimated at 7.5 Gb [23,24] and is expected to be largely made up of repeat elements. Therefore, sequencing the gene space is likely to represent a several hundred-fold reduction in complexity. In this study, we sequenced, assembled, and characterised the normalised leaf transcriptomes of two *Ae* *. sharonensis* accessions to identify 62,243 non-redundant expressed sequences. Using the reads from individual accessions we predict 8,904 sequences that carry SNPs. These resources can be used to map traits of interest in *Ae* *. sharonensis* , aid in the reconstruction of the evolutionary origins of the three genomes of hexaploid bread wheat, and delimit the preferentially introgressed *Gametocidal* gene-containing translocations in wheat [4].

## Results and Discussion

### Sequencing of the* Ae sharonensis* leaf transcriptome

Two *Ae* *. sharonensis* accessions were chosen based on their reaction to Ug99 (race TTKSK), a highly virulent isolate of *Puccinia* *graminis* f. sp. *tritici* (*Pgt*), the causal agent of wheat stem rust. Accession 1644 is resistant to *Pgt* race TTKSK while accession 2232 is susceptible [6]. Total RNA was extracted from pooled tissue of the 2^nd^, 3^rd^, and 4^th^ leaves of the two accessions, cDNA synthesised, normalised, 454 sequencing libraries prepared, and sequenced on two full plates (one for each accession) of the 454 GS-FLX sequencer with Titanium chemistry (see Methods). The initial sequencing produced 1,338,956 reads (797 Mbp) for accession 1644 and 1,355,371 reads (852 Mbp) for accession 2232. The data were processed by clipping off the sequencing adapters and removing low quality reads leaving 1,337,801 reads for 1644 and 1,354,518 reads for 2232 as our initial dataset. Files containing the initial set of clipped reads have been deposited in the Sequence Read Archive (SRA) accessible via www.ebi.ac.uk/ena/data/view/ERP001258. In addition, the dataset was curated to remove reads below 45 nucleotides, known *Triticeae* repeat sequences, and chloroplast, mitochondrial, and potential human contamination (see Methods). The numbers of reads after each processing step are presented in Table 1. We used 1,247,583 reads from accession 1644 and 1,280,912 from accession 2232 as the input data for *de novo* transcriptome assembly.

**Table 1:** Preprocessing of 454 transcriptome reads.

**Step^a^**	**Accession 1644**	**Accession 2232**
Raw reads	1,338,956	1,355,371
Clipped reads	1,155 (-0.08%)	853 (-0.06%)
Reads ≤ 45nts	62,233 (-4.65%)	54,918 (-4.05%)
Triticeae repeats	10,850 (-0.85%)	9,001 (-0.69%)
Organelle DNA	16,586 (-1.31%)	9,427 (-0.73%)
Human repeats	459 (-0.04%)	260 (-0.02%)
Total	1,247,583	1,280,912

^a^ Number of 454 sequencing reads obtained after sequencing and each consecutive quality control and pre-processing step.

### Transcriptome assembly

**Figure 1:**
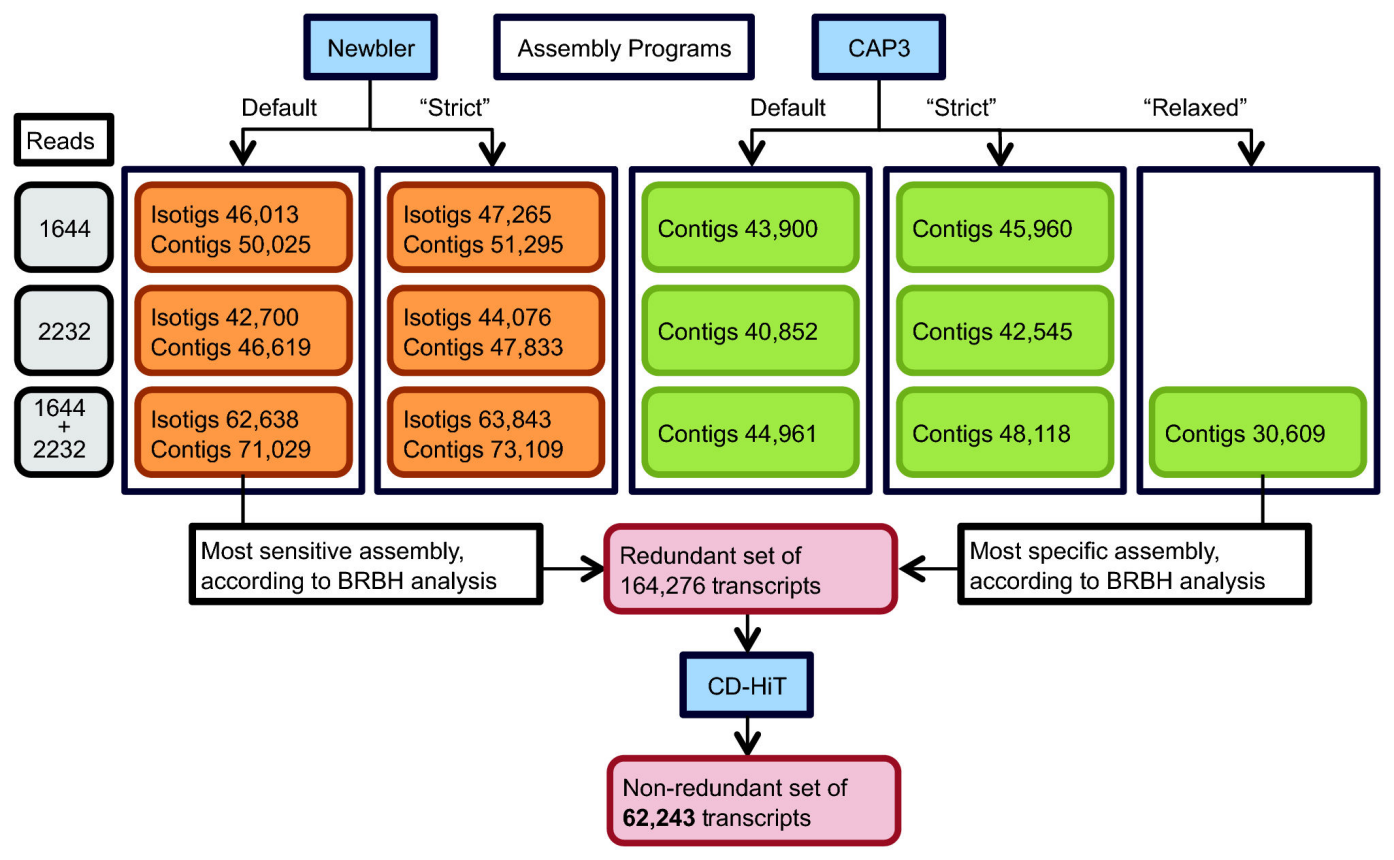
Strategy to obtain extended representation of the expressed *Ae* *. sharonensis* leaf gene space. Overview showing the sequence input (far left), assembly program (Newbler or CAP3; top), and parameters (default, strict or relaxed; see Methods), for each assembly, and extraction of a non-redundant sequence set.

Studies comparing various *de novo* assembly programs found that the overlap-layout-consensus assemblers CAP3, designed for assembling ESTs [25], and Newbler 2.5.3, Roche’s 454 GS assembler [26], work as good as or better than other assemblers for 454 data sets [27–29]. We employed both the CAP3 and Newbler assembly programs to obtain a more extended representation of the expressed gene space in the leaf [28]. The Newbler assembler reports together with the set of contigs a set of isotigs in an attempt to represent different spliced variants of the same genomic loci (see Methods). For each Newbler assembly performed in this study we report both the contig and the isotig sets. Three different groups of assemblies were performed, two by using reads from each accession individually and one group of assemblies where all the reads from both the accessions were used in combination. Each assembly program was used, for each of the three different assembly groups, with the default as well as a set of custom ‘strict’ parameters resulting in 12 different *de novo* assemblies of the *Ae* *. sharonensis* leaf transcriptome. One additional CAP3 assembly with ‘relaxed’ parameters was performed using identical parameters to the ones that generated the barley unigene35 assembly [13], an assembly that represents a wide sampling of the barley gene space. For an overview of the assemblies, see Figure 1. Technical details of the assembly procedures are described in Methods, and statistics of the contigs/isotigs of the 13 assemblies are summarised in Table 2. The Newbler and CAP3 assemblies using the ‘combined’ dataset produced 35-43% and 2-13% more contigs than the single accession assemblies, respectively, and were used as pseudo-reference sequences for detection of SNPs. The individual assemblies of each accession were used for studying particular gene families.

**Table 2:** Assembly statistics.

**Assembly**		**N50^a^**	**Longest Sequence**	**Assembly Size^b^**
Combined Newbler Default	Isotigs 62,638	1,046	12,183	47,260,382
	Contigs 71,029	674	12,183	36,319,190
Combined Newbler Strict	Isotigs 63,843	875	11,149	42,344,706
	Contigs 73,109	657	11,149	36,621,727
Combined CAP3 Default	Contigs 44,961	688	3,611	27,827,460
Combined CAP3 Strict	Contigs 48,118	640	5,119	28,444,221
Combined CAP3 Relaxed	Contigs 30,609	815	6,013	22,922,091
1644 Newbler Default	Isotigs 46,013	950	12,182	32,907,417
	Contigs 50,025	716	12,182	27,980,764
1644 Newbler Strict	Isotigs 47,265	832	10,210	30,789,093
	Contigs 51,295	690	9,046	27,986,055
1644 CAP3 Default	Contigs 43,900	639	4,441	26,481,168
1644 CAP3 Strict	Contigs 45,960	612	6,754	26,661,948
2232 Newbler Default	Isotigs 42,700	723	9,194	26,017,733
	Contigs 46,619	638	5,090	23,332,425
2232 Newbler Strict	Isotigs 44,076	688	7,373	25,070,068
	Contigs 47,833	624	7,373	23,346,945
2232 CAP3 Default	Contigs 40,852	616	3,730	23,156,778
2232 CAP3 Strict	Contigs 42,545	597	3,729	23,343,285

^a^ The N50 is defined as the length of the contig that spans the median length of the full assembly.

^b^ Nucleotides

To assess the reliability of each assembly we correlated the length of each individual contig (or isotig where available) against the number of reads that generated this contig [30]. The plots we obtained (Figure S1) are expected plots of normalised mRNA and comparable among all the different assemblies. The length of contigs (or isotigs) and the number of reads that constructed each contig are correlated indicating that each assembly is reliable. An additional approach to assess the reliability of the assemblies is to align each set of contigs (or isotigs) against itself using BLASTn [31]. With this approach chimeric, repeated, and erroneously mated contigs will appear as high hits in the BLASTn results. The total number of significant self-BLASTn hits reveals the degree of redundancy in the contig/isotig datasets and provides an additional indication of the reliability of the assembly. For our assemblies this proportion varied between 1.08% and 1.55% for all the 13 contig sets and between 1.27% and 2.03% for the six isotig sets. These proportions represent repeated domains and/or a very limited number of chimeric sequences generated in our transcriptome assemblies.

The quality of the different assemblies of the *Ae* *. sharonensis* leaf transcriptome was assessed by analysing the orthologous relationships with the gene space of related grass species. We used the predicted protein coding genes from four sequenced and annotated grass genomes including rice [14], *Sorghum* [15], maize [16] and *Brachypodium* [12], in addition to available barley *Unigenes* [13] and the combined set of Triticeae (wheat and barley) full-length cDNAs [17]. Contigs from all the assemblies were aligned against the above-mentioned predicted proteomes by BLASTx (and BLASTn for the cDNAs) and the reciprocal hits were obtained by tBLASTn (and tBLASTx for the cDNAs). The Best Reciprocal Blast Hits (BRBH) from each assembly were calculated and presented in Table 3 (see Methods for details). The grass gene sequence set that exhibited the largest proportion of BRBH with *Ae* *. sharonensis* is the wheat/barley (*Triticeae*) full-length cDNA set and this was expected because *Ae* *. sharonensis* , as a member of the *Sitopsis* section of *Aegilops* [32] belongs to *Triticeae*. However, the largest number of BRBH came from the next closest relative, barley. This can be explained because the barley unigene set contains more than three times the number of sequences present in the Triticeae full-length cDNA set and is considered highly representative of the transcriptome across all tissues.

**Table 3:** Best reciprocal BLAST hits of *Ae* *. sharonensis* transcripts against barley unigenes, wheat full-length cDNAs and the exomes of sequenced grasses.

**Assembly**		**Barley35**	**TriFLDB**	** *B* *. distachyon* **	***O. sativa***	***S. bicolor***	***Z. mays***	**Unique**
		**50,938**	**15,871**	**32,255**	**51,258**	**29,448**	**106,046**	**Hits^1^**
Combined Newbler Default	Isotigs	15,329	6,110	10,254	9,792	9,687	9,618	18,871
	Contigs	15,785	6,015	10,047	9,641	9,553	9,506	18,684
Combined Newbler Strict	Isotigs	15,447	6,083	10,154	9,717	9,640	9,574	18,454
	Contigs	15,800	6,020	10,044	9,610	9,548	9,518	18,351
Combined CAP3 Default	Contigs	13,650	5,642	9,159	8,774	8,695	8,708	16,606
Combined CAP3 Strict	Contigs	13,675	5,627	9,177	8,756	8,649	8,701	16,715
Combined CAP3 Relaxed	Contigs	13,464	5,254	9,185	8,788	7,433	8,702	15,687
1644 Newbler Default	Isotigs	14,249	5,748	9,345	8,930	8,856	8,892	16,886
	Contigs	14,552	5,699	9,253	8,848	8,791	8,849	16,941
1644 Newbler Strict	Isotigs	14,345	5,746	9,287	8,881	8,814	8,846	16,908
	Contigs	14,577	5,703	9,221	8,827	8,769	8,795	16,998
1644 CAP3 Default	Contigs	13,511	5,379	8,475	8,170	8,132	8,257	16,451
1644 CAP3 Strict	Contigs	13,440	5,367	8,438	8,157	8,112	8,197	16,454
2232 Newbler Default	Isotigs	14,645	5,712	9,529	9,086	8,979	8,990	17,395
	Contigs	14,758	5,692	9,480	9,040	8,942	8,937	17,294
2232 Newbler Strict	Isotigs	14,644	5,700	9,496	9,041	8,942	8,982	17,371
	Contigs	14,735	5,693	9,454	9,015	8,913	8,927	17,337
2232 CAP3 Default	Contigs	13,190	5,482	8,951	8,512	8,446	8,431	15,975
2232 CAP3 Strict	Contigs	13,174	5,484	8,940	8,542	8,438	8,460	16,033

1 Unique hits refer to the non-redundant sequences from the union of all best reciprocal BLAST hits from all six datasets.

**Figure 2:**
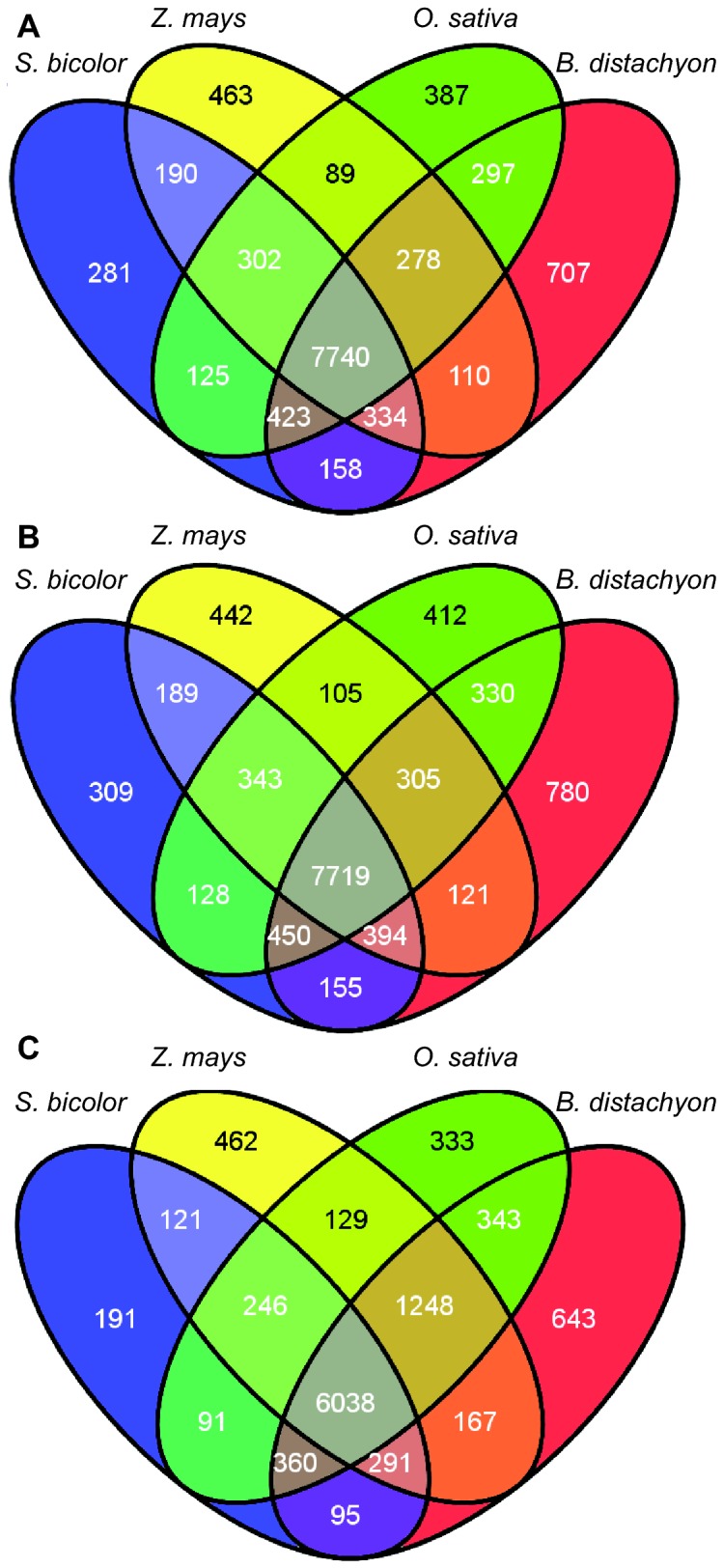
The overlap of *Ae* *. sharonensis* best reciprocal BLAST hits against four grass proteomes. (A) Contigs of the Newbler default assembly. The core set of 7,740 common orthologs of *Ae* *. sharonensis* among all grasses represents 35.6% of all four grass orthologs for this assembly. (B) Isotigs of the Newbler default assembly. The core set of 7,719 common orthologs represents 63.3% of all the four grass orthologs for this assembly. (C) Contigs of the ‘CAP3 relaxed’ assembly. The core set of 6,038 common orthologs represents 56.1% of all the four grass orthologs for this assembly.

The coverage of the grass gene space for each assembly was calculated by counting the unique hits from each assembly contig set that are BRBH with all the grass genes (Table 3). This number constitutes a measure of how many orthologous sequences each assembly has captured and is a proxy for the breadth of each assembly. Different assemblies of the same dataset generated qualitatively different coverage of the gene space: the Newbler assembler using default parameters produced the most unique BRBH against all the grass gene sets. Newbler generated a contig set that spans the grass gene space more than any other assembly (18,871 unique BRBH) and CAP3 a contig set with the highest proportion of unique BRBH (more than 50% of the CAP3 relaxed contigs have a unique BRBH; 15,703 out of 30,609 contigs) (Table 3). Furthermore, a similar result was observed when we limited the BRBH to the four fully annotated grass predicted proteomes and focused on a “core” set of BRBH that are shared among all of the four grasses; Figure 2 illustrates the relationships between the BRBH of the Newbler default assembly contigs (Figure 2A), isotigs (Figure 2B) and the CAP3 relaxed assembly contigs (Figure 2C). Newbler default contigs capture a high number (7,740) of the “core” orthologs of the four grasses while CAP3 relaxed contigs capture the most “specific” set (6,038) of the core orthologs. The latter is illustrated by the high number of ‘core’ BRBH of the CAP3 assembly (1,248 hits) if *Sorghum* is excluded compared to the Newbler assemblies (278 and 305 hits); as *Sorghum* is the least well annotated grass proteome the CAP3 assembly has excluded many of the more remote orthologs from the BRBH analysis. The intersections of these sets of BRBH between *Ae*. *sharonensis* and the four grass proteomes constitute a core collection of orthologs of *Ae* *. sharonensis* transcripts and are suitable to be used for grass comparative genomics studies.

In conclusion, combining reads from both accessions led to assemblies with a higher number of longer contigs as well as BRBH with other grasses. However, the *de novo* assemblies of the *Ae* *. sharonensis* leaf transcriptome demonstrated that overall none of the assembly programs or parameter sets clearly performed better than the others in the BRBH analysis. For further ortholog analysis we used the sequences generated by two of the 13 assemblies: (i) the combined Newbler default assembly, chosen for its ability to separate paralogs (i.e. it generated the highest number of contigs and the highest coverage of the grass gene space), and (ii) the combined CAP3 relaxed assembly, chosen for its power to place sequence variants in the same contig, resulting in a shorter yet more accurate representation of the transcriptome (i.e. it provided the highest proportion of BRBH with grass genes with the lowest number of contigs). The three sequence sets (two sets of contigs and one of isotigs) were used for the functional analysis of the transcriptome and to extract a non-redundant sequence set from the *Ae* *. sharonensis* leaf transcriptome.

### *Ae sharonensis* non-redundant sequence set

The contigs and isotigs from the most sensitive assembly (the Newbler default assembly) as well as the contigs from the most specific assembly (the CAP3 relaxed assembly) were clustered in a set of non-redundant sequences using CD-HIT (Figure 1; see Methods). CD-HIT clusters similar sequences together and reports the longest representative sequence from each cluster. Out of a total of 164,276 contig and isotig sequences from the three assemblies, 62,243 were reported back as a non-redundant set and constituted the non-redundant sequence collection of the *Ae* *. sharonensis* transcriptome (Figure 1).

### Functional characterisation of the *Ae sharonensis* leaf transcriptome

**Figure 3:**
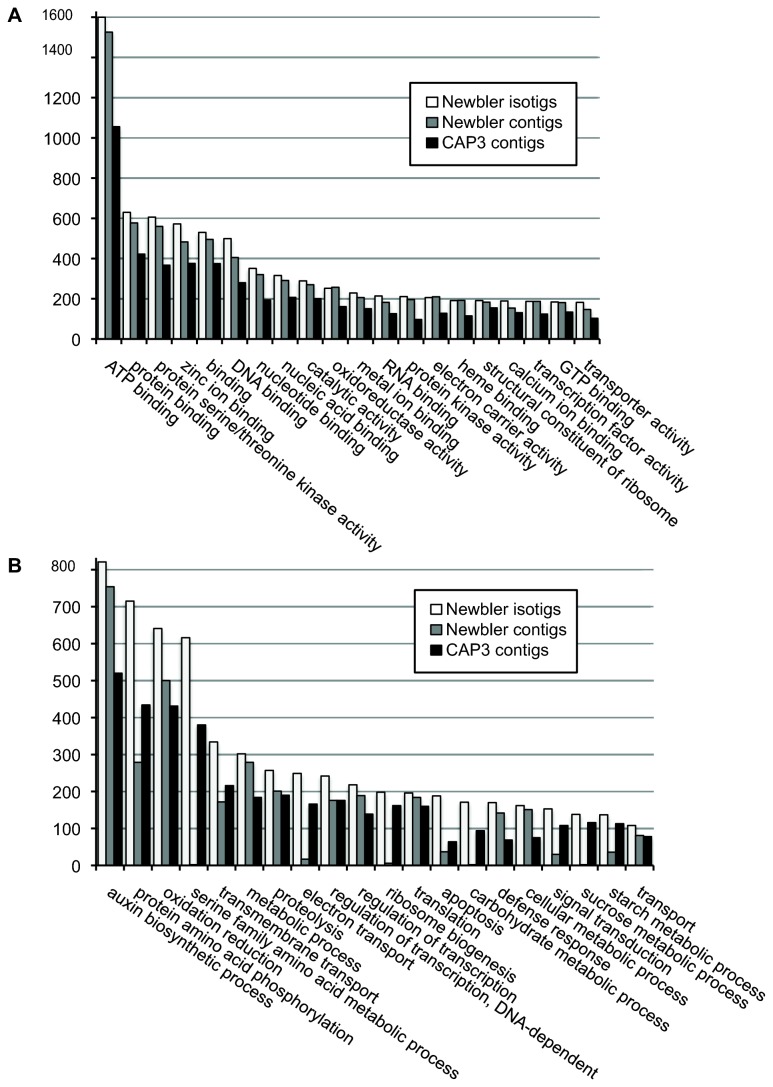
GO molecular function categories hits. Frequencies of contig/isotig hits in the top 20 GO term categories from two *Ae* *. sharonensis* transcriptome assemblies (contigs and isotigs from the Newbler default assembly and contigs from the CAP3 relaxed assembly). (A) Molecular Function categories. (B) Biological Processes categories.

Functional annotation of the assembled transcripts was conducted in two rounds. First, all contigs/isotigs from the two selected *Ae* *. sharonensis* assemblies (Newbler default and CAP3 relaxed) were aligned against the NCBI non-redundant protein database (nr) for functional characterisation and to search for potential contamination of the RNA samples with those of other species. The level of BLASTx hits against the nr that did not have a plant species as a top hit was found to be 0.6% on average in all the combined assemblies (Table S1). Furthermore, 97% of the BLASTx hits came from grass species (namely rice, *Sorghum* , maize, wheat and barley) consistent with the reads as well as the assembled contigs having indeed derived from a grass species (Table S1). Second, contigs from the two selected assemblies were aligned against the UniRef50 protein database of UniProt for the assignment of Gene Ontology (GO) terms to individual transcripts. GO terms were assigned for every contig that exhibited a significant hit. The numbers of hits for the top 20 molecular function GO term categories for the two assemblies are illustrated in Figure 3A and for the top 20 biological processes in Figure 3B.

To complete the functional characterisation of the transcriptome, a Pfam scan (see Methods) was performed on the contigs/isotigs of each of the Newbler default and CAP3 relaxed combined assemblies to identify protein family profile hits. The Pfam scan identified 21,808 hits in the Newbler default isotigs, 19,539 hits in the contigs set and 14,762 hits in the CAP3 relaxed assembly (Additional file 4).

### Resistance gene homologues

We analysed the GO “biological function” category to search for proteins related to plant immunity (see Methods). The nucleotide binding leucine-rich repeat gene family (NB-LRR) is involved in the resistance of plants against biotic stress and is under strong diversifying selection [33]. The genetic mechanisms that generate variation in this family include unequal crossing over (leading to tandem duplications), gene-conversion, and point mutation [34,35]. Through diversifying selection, as a result of the evolutionary pressure exerted by pathogens, NB-LRR family genes exhibit high variability in the LRR domain, and relatively higher levels of similarity in the core nucleotide binding-site, the so-called NB-ARC domain [36]. As the NB-LRR gene family is fast-evolving and exhibits rapid gene expansion [37], we hypothesised that some NB-LRR family members would not be detected by the GO terms search above. Therefore, we performed a Hidden Markov Model (HMM) search to identify novel NB-LRR genes. We used all the available assemblies of the *Ae* *. sharonensis* transcriptome to predict NB-ARC domain containing contigs using the Pfam NB-ARC HMM profile. Subsequently, we searched the predicted NB-ARC contigs for sequences that contain an intracellular LRR clan HMM profile to identify novel NB-LRR gene family members (see Methods). For each predicted set of NB-LRR genes we then calculated the BRBH against all the NB-LRRs from four grass species with sequenced genomes [38] (Table 4). The differences in the numbers of assembled NB-LRRs between different assemblies (in contrast to other gene families as evidenced by the BRBH study) demonstrate the difficulty of reconstructing the full complement of this complex multi-gene family from these datasets.

**Table 4:** Predicted NB-LRR proteins from *Ae* *. sharonensis* assemblies and BRBH against grass NB-LRRs.

		**Predicted domain**		**BRBH to grass NB-LRRs**	
**Assembly**		**NB-ARC**	**NB-LRR**	**NB-ARC**	**NB-LRRs**
Combined Newbler Default	Isotigs	443	86	99	33
	Contigs	398	41	102	30
Combined Newbler Strict	Isotigs	435	60	102	32
	Contigs	410	40	106	28
Combined CAP3 Default	Contigs	161	4	62	3
Combined CAP3 Strict	Contigs	164	4	55	3
Combined CAP3 Relaxed	Contigs	144	4	62	4
1644 Newbler Default	Isotigs	312	53	85	30
	Contigs	296	36	88	27
1644 Newbler Strict	Isotigs	313	38	87	26
	Contigs	305	31	89	25
1644 CAP3 Default	Contigs	236	11	72	10
1644 CAP3 Strict	Contigs	243	8	73	7
2232 Newbler Default	Isotigs	194	21	67	8
	Contigs	182	10	69	8
2232 Newbler Strict	Isotigs	178	7	66	7
	Contigs	176	7	68	7
2232 CAP3 Default	Contigs	130	4	48	3
2232 CAP3 Strict	Contigs	132	2	53	2

**Figure 4:**
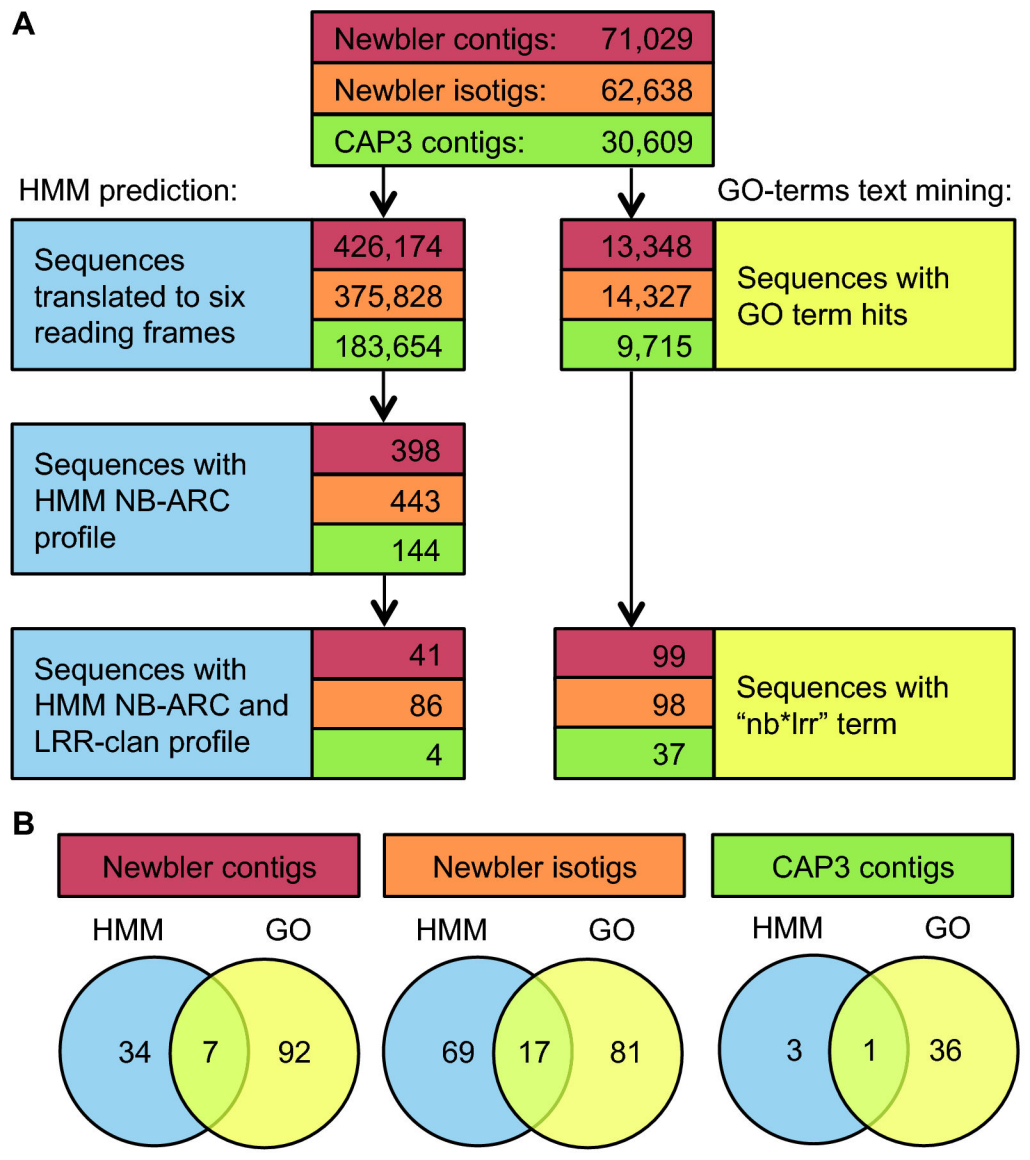
Analysis of the prediction approaches for members of the NB-LRR gene family. (A) Flowcharts of the two NB-LRR prediction approaches; HMM based left, BLAST and GO terms right. (B) Venn diagrams depicting overlaps between the two approaches.

By searching in the GO term results for NB-LRRs (see Methods), we identified 99 contigs and 98 isotigs from the Newbler default assembly, and 37 contigs from the relaxed CAP3 assembly. Out of the 99 Newbler contigs only seven were also identified by the HMM approach (out of a total of 41 predicted by HMM). For the Newbler isotigs, 17 were also identified by HMM (out of a total of 86 predicted by HMM). For the CAP3 relaxed assembly, out of the 37 contigs with an associated NB-LRR GO term only one was in common with the four contigs that the HMM search identified (Figure 4). This narrow overlap highlights the diversifying nature of the NB-LRR gene family, supporting our hypothesis that homology-based search alone cannot predict the full complement of NB-LRRs, and suggesting that a combined approach which includes BLAST homology search (which predominantly identifies already sequenced NB-LRRs) as well as an HMM search (which will detect some novel NB-LRRs) allows access to a greater coverage of the NB-LRR sequence space in a species with an uncharacterised genome.

### Prediction and characterisation of SNPs between the two *Ae sharonensis* accessions

Transcriptome sequencing of two different accessions of *Ae* *. sharonensis* (1644 and 2232) provides a means for the prediction of SNPs between expressed genes of the two accessions. We used the contigs and isotigs from the combined *de novo* ‘Newbler default’ assembly, as pseudo-references to align the reads from the two accessions using the Mosaik pipeline. SNPs were called using GigaBayes, which uses a Bayesian framework to predict SNPs (see Methods for full description of the pipeline).

The Mosaik/GigaBayes pipeline predicted 16,227 SNPs in isotigs and 18,195 SNPs in contigs of the Newbler default assembly. Furthermore, 14,733 SNPs were identified within the 18,684 grass BRBH of the *Ae* *. sharonensis* contigs and 13,353 within the 18,871 BRBH of isotigs of the Newbler default assembly. These SNPs will facilitate the generation of genetic markers to syntenic regions in other characterised monocot genomes. Therefore these SNPs are particularly useful for mapping traits in *Ae* *. sharonensis* and for comparative genomic studies between grasses. Of the 62,243 non-redundant sequences identified in *Ae* *. sharonensis* 24,656 contained one or more SNPs totalling 51,610 SNPs, as predicted by the Mosaik/GigaBayes pipeline (Table S2). The number of predicted SNPs from each assembly as well as SNPs in grass BRBH and UniRef50 hits are summarised in Table 5 and the complete sets of SNPs within their sequence context are available as a General Feature Format (GFF) file in Text S1.

**Table 5:** SNPs predicted between the two *Ae* *. sharonensis* accessions from two selected assemblies.

	**Total SNPs**	**SNPs in BRBH^a^**	**SNPs in UniRef50 hits**
Combined Newbler Default Contigs	18,195	14,733	16,600
Combined Newbler Default Isotigs	16,277	13,353	14,923

^a^ Those SNPs occurring in contigs and isotigs that have a BRBH in any of the six grass proteomes.

The marker conversion rate was estimated for a subset of SNPs identified between 1644 and 2232. Of the 59 SNPs tested, 35 (59%) were successfully converted into co-dominant markers. No difference was observed between marker conversion rates for “Total SNPs” (17 of 27 SNPs) and “SNPs from BRBHs” (18 of 32 SNPs). Failure to convert into a marker can be associated with incorrectly predicted exon-intron boundaries, interference from other polymorphisms in primer sites, or the assembly of paralogs. Thus, the estimated marker conversion rate of 59% should be considered an underestimate of the true degree of polymorphism between 1644 and 2232 detected in this SNP pipeline.

The high number of SNPs between these two accessions from two geographically distinct populations, 1644 from Ashdod in the Philistine Plain, southern Israel, and 2232 from En HaMifraz in northern Israel [6], is consistent with the high degree of genetic diversity found within and between *Ae* *. sharonensis* populations, as revealed by a previous study using 21 SSR markers on 106 accessions [9].

### Heterozygosity in *Ae sharonensis* accessions 1644 and 2232

**Figure 5:**
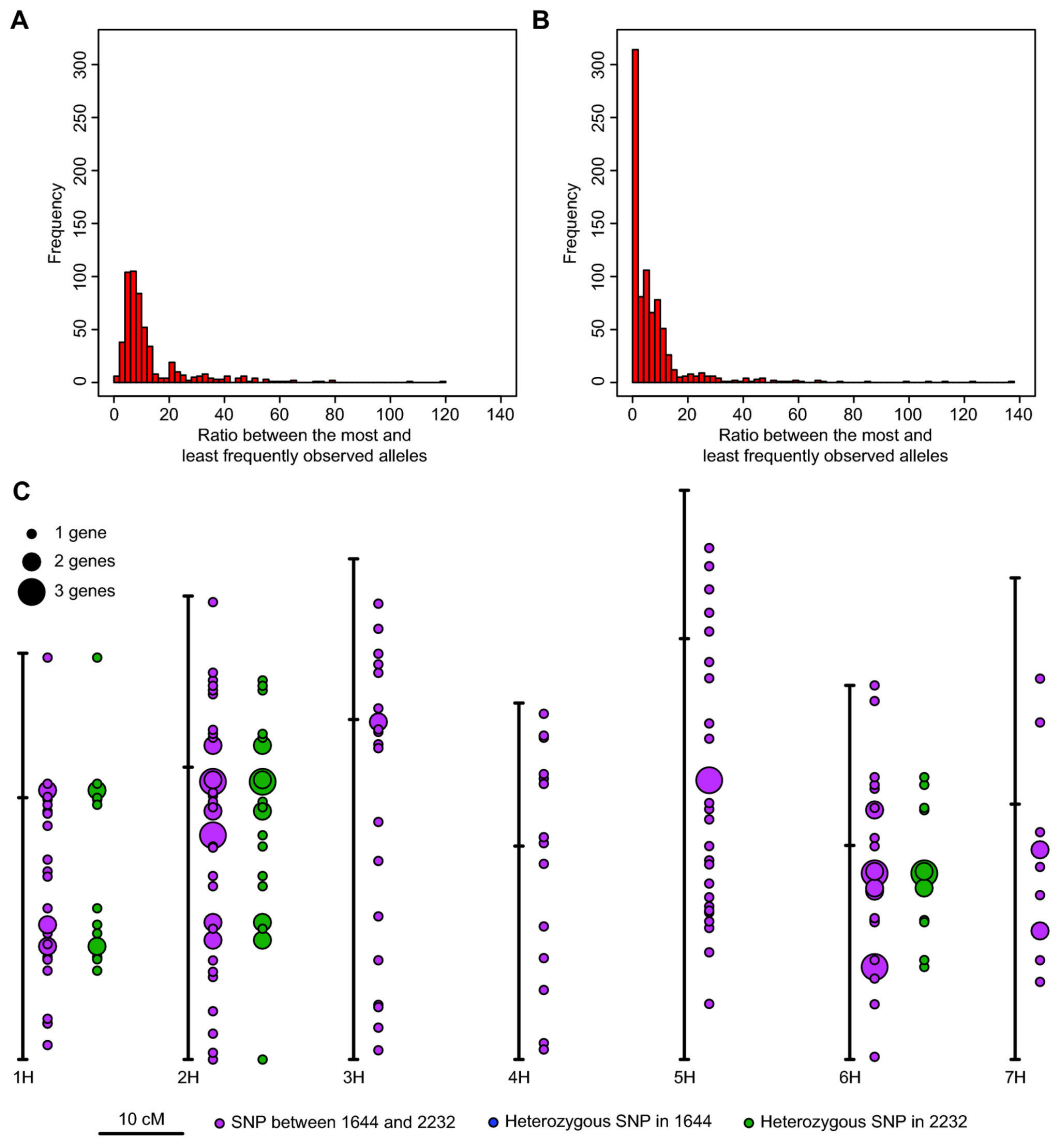
Analysis of heterozygosity in *Ae sharonensis* accessions 1644 and 2232. Histograms of the ratio between the most and least frequently observed alleles of individual SNP with read coverage of ≥20 between accessions (A) 1644 and (B) 2232 using the ‘Combined Newbler Default Isotigs’. Heterozygous SNP-bearing *Unigenes* were identified using a ratio threshold of 3.0 (most versus least frequently observed allele within a genotype) and mapped using best reciprocal BLAST hits to genes in the barley consensus genetic map (C). Dot size reflects number of SNPs in a genetic bin (see legend).

Although *Ae* *. sharonensis* is generally considered autogamous, outcrossing is fairly common [39]. The rich source of SNPs provided a basis with which we could determine the degree of homo- and heterozygosity in accessions 1644 and 2232. Examination of the ratio between the most- and least-frequently observed alleles of individual SNPs with read coverage of ≥20 (444 SNP-bearing isotigs from the combined Newbler default assembly) found significantly different histograms between accessions 1644 and 2232 (Figure 5A and 5B), respectively. Using an allelic ratio cutoff of less than or equal to 3.0 (range of 1:1 to 3:1), 7 and 136 isotigs contained a ~1:1 mixture of alleles of the SNP in accessions 1644 and 2232, respectively. Similar results were observed for the contig assemblies generated by Newbler and CAP3 (data not shown). This suggests an overall heterogeneity of 1.6% in accession 1644 and 30.1% in accession 2232, respectively. After collection in the wild, 1644 and 2232 were maintained as inbred lines for one generation under controlled glasshouse conditions. Thus, the heterozygosity observed in accession 1644 reflects either true residual heterozygosity from the wild or paralogs that have been placed into the same isotig. In contrast, accession 2232 was either highly heterogeneous when isolated in the wild or outcrossed in the greenhouse during the selfing process.

Mapping of heterozygous SNPs onto a genetic map would identify the regions still heterozygous in accession 2232 and would provide support for their authenticity. In lieu of an *Ae*. *sharonensis* genetic map, we mapped the SNP-bearing isotigs onto the consensus barley genetic map [11] based on BRBH (*e*-value cutoff 10^-10^). Of the 444 SNP-bearing isotigs identified, 180 isotigs had putative orthologs that had been mapped in barley (Figure 5C). Of these, none were heterozygous SNPs from 1644 and 59 from 2232 were placed on the barley genetic map (Figure 5). Strikingly, the SNP-bearing isotigs of 2232 co-localized to chromosomes 1H, 2H and 6H. This suggests that barley and *Ae* *. sharonensis* share a reasonable degree of synteny and that these SNPs within 2232 are due to a recent cross-pollination event between two distinct accessions. The high degree of homozygosity of 1644 makes this accession an ideal candidate reference accession for the generation of future molecular genomics tools such as whole genome sequencing and BAC library construction.

## Conclusions

We present the *de novo* assembly, the functional characterisation, and the genetic variability of the leaf transcriptome of two *Ae* *. sharonensis* accessions. By comparing the results of different assembly programs and different parameters we assess the quality and breadth of each transcriptome assembly based on orthology detection with other *Poaceae* species. We conclude there is not a “single” assembly that outperforms the rest and the use of each assembly is relevant to the biological question at hand. By using a simple BRBH strategy we readily assess, validate and compare the results from our selected assemblies with other grass species and by clustering the contigs/isotigs of the selected assemblies we report a set of non-redundant *Ae* *. sharonensis* leaf transcriptome sequences. We predict SNPs in the assembled expressed genes for this species with an uncharacterised genome and establish a conversion rate of 59% of the SNPs into co-dominant markers in a Sequenom genotyping assay. The transcriptome assemblies, the core ortholog set, and the SNPs we have predicted from the *Ae* *. sharonensis* transcriptome provide a valuable resource to clone traits of agronomic interest from this wild wheat relative, delimit *Gametocidal* loci, further characterise the cereal gene space, and help elucidate the relationship of *Ae* *. sharonesis* with hexaploid wheat.

## Materials and Methods

### Plant germplasm

*Ae* *. sharonensis* accessions 1644 and 2232 [6], have been self fertilized once under controlled conditions (Pablo Olivera, personal communication). Seeds were germinated and grown to the four-leaf stage in a growth cabinet. Tissue was harvested from single plants of 1644 and 2232, which were then vernalised and grown to maturity for seed production.

### *Ae sharonensis* transcriptome sequencing

Total RNA was extracted from leaf tissue obtained from the second to fourth leaves of *Ae* *. sharonensis* accessions 1644 and 2232, and treated for removal of DNA with DNAase. Poly(A)^+^ RNA was isolated and used as a template for first-strand cDNA synthesis using an N_6_ randomized primer. The cDNA was amplified with 12 PCR cycles using the proof reading polymerase Herculase II (Agilent Technologies, Santa Clara, CA). cDNA normalization was performed by one cycle of denaturation and reassociation followed by passing the mixture over a hydroxylapatite column to separate the reassociated ds-cDNA from the remaining (normalised) ss-cDNA. The ss-cDNA was then amplified with 10 PCR cycles. The normalised cDNA was analysed and size-fractionated on a Shimadzu MultiNA microchip electrophoresis system. The cDNA was eluted from the preparative agarose gels in the size range of 600 to 800 bp. 454 Life Sciences sequencing adaptors A and B were ligated to the 5’ and 3’ ends of the cDNA, respectively, to construct two 454 sequencing libraries; one for each accession. The cDNA from the two accessions was sequenced on a 454 Genome Sequencer FLX Titanium Series platform (Roche Diagnostics) on one full sequencing plate for each accession. Raw reads were processed to remove sequencing adaptors and low quality reads and deposited in the Sequence Read Archive (SRA) accessible via www.ebi.ac.uk/ena/data/view/ERP001258. cDNA synthesis, normalisation, library preparation, sequencing, adaptor clipping and initial quality control of the raw reads were performed by Eurofins MWG Operon, Ebersburg, Germany.

### Preprocessing of 454 reads

The maximum read length of the clipped reads was 828 nts, which is not significantly higher than the expected upper read length range (600-800 nts). Therefore no long read removal was considered necessary. The clipped 454 cDNA reads were size-selected for reads longer than 45 nts. The reads were treated for the removal of Triticeae specific repeat sequences, which occur in cDNA from transcriptionally active retrotransposons. The BLAST+ suite for sequence alignment [40] version 2.2.25 obtained from the National Center for Biotechnology Information (NCBI) was used to conduct every sequence alignment in this work. Reads that matched a repeat from the Triticeae Repeat Sequence (TREP) database (http://wheat.pw.usda.gov/ITMI/Repeats/; accessed on 15 July 2011) by BLASTn (e-value ≤ 10^-6^, alignment ≥ 85% of the query and identity ≥ 85%), were removed. This conservative cutoff reduces the likelihood of falsely removing non-transposon transcribed genes. The transcriptional machinery in organelles can generate long transcripts. To remove organelle DNA from the *Ae* *. sharonensis* reads we used the mitochondrial and chloroplast genome sequences of wheat, as wheat is the closest species with fully sequenced organelle genomes. Reads that aligned with the wheat organelles’ DNA by BLASTn (e-value ≤ 10^-6^, alignment ≥ 90% of the query and identity ≥ 90%), were removed. A recent report demonstrated the contamination of sequence databases by human DNA [41]. To remove potential human contamination from the *Ae* *. sharonensis* 454 reads, human specific repetitive sequences were retrieved from the RepBase (version 16.02) [42], (http://www.girinst.org/repbase/index.html) and reads that aligned against the repeats by BLASTn (e-value ≤ 10^-6^, alignment ≥ 90% of the query and identity ≥ 90%) were removed.

Analysis of the remaining sequences showed that the number of nucleotides that were ambiguously called by the sequencer (Ns) was 0.14% of the total number of nucleotides, and 91.9% of the Ns occurred in the last half of the read [26].

### Assembly of the *Ae sharonensis* transcriptome

Two assembly programs based on the Overlap Layout Consensus (OLC) approach were used for the *de novo* assembly of the 454 reads; the EST assembler software CAP3 [25], and the 454 Roche GS-FLX *de novo* assembler (Newbler 2.5.3) [26] obtained from (http://my454.com/contact-us/software-request.asp). This version of Newbler introduces the -urt option, which bridges contigs across regions with only single-depth coverage [28]. We used the -cdna option of Newbler, which, after the assembly of contigs, transverses the graph of contigs extentions to generate spliced variants (isotigs) for each clustered set of contigs. Each assembly program was run with the default parameters as well as with a set of empirically defined stringent parameters, referred to as ‘strict’. Both ‘default’ and ‘strict’ Newbler assemblies were performed using the -cdna flag which together with the set of contigs reports a set of isotigs. An isotig is defined as a predicted transcript variant from a certain locus. We kept and analysed both the contigs as well as the isotigs. The ‘strict’ Newbler parameters were set as follows: -icl 50 -icc 30 -ig 150 -ml 50 -mi 95, while the ‘strict’ CAP3 parameters were specified as: -p 95 -d 60 -f 100 -h 50 (http://www.harvest-web.org/). In addition to the default and ‘strict’ CAP3 assemblies for the combined set of reads we performed an assembly using parameters identical to those used in a less stringent assembly of barley (-p 75 -d 200 -f 250 -h 90). This assembly, named ‘relaxed’ in our analysis, was performed to allow a direct comparison of assemblies between these two different organisms.

BLAST e-value thresholds for the Best Reciprocal BLAST Hits (BRBH) were set to 10^-10^. BLASTx was used for the alignments of contigs to the grass proteins and tBLASTn for the reciprocal. The protein sets of the fully sequenced grasses *Oryza sativa, Zea mays,* *Brachypodium* *distachyon* and *Sorghum* *bicolor* were obtained from the predicted proteomes in the Phytozome database (http://www.phytozome.net/). The Triticeae (wheat and barley) full-length cDNAs are the non-redundant set of full-length CDS from RIKEN (http://trifldb.psc.riken.jp/download.pl) and the barley *Unigenes* are the ‘relaxed’ barley assembly 35 (http://www.harvest-web.org/). The BRBH algorithm was implemented using an in-house Perl script. To demonstrate that the generation of cDNA (see sequencing methods section above) is a product of random priming and no substantial positional bias was introduced in the generation of 454 reads, the nucleotide coverage at each individual position of the contigs of the Newbler ‘default parameters’ assembly was calculated. Figure S2 includes boxplots for each individual contig position (relative to the sequence length) for every contig >100 nucleotides. The median coverage at each position does not show any substantial positional bias towards the 3’ UTR and is congruent with the random priming generation of the cDNA.

### Identification of non-redundant sequence set

The CD-HIT program [43] for clustering nucleotide sequences was used to identify the non-redundant sequence set. CD-HIT groups similar nucleotide sequences into clusters that meet a similarity threshold. We checked the robustness of the retrieved sequences with 70, 80, 90, 95 and 98% similarity, before choosing the default 90%. All the rest of the parameters were left to the default values.

### Transcriptome functional analysis

For the functional analysis and characterisation of the assemblies two protein databases were used. The non-redundant protein sequence database from NCBI (nr) (ftp://ftp.ncbi.nih.gov/blast/db/; downloaded in April 2011) was used for the species characterisation of contigs and the validation of the *Ae* *. sharonensis* leaf transcriptome assembly, as was the UniRef50 [44] (http://www.uniprot.org/help/uniref; accessed on 17 June 2011), which is a version of the UniRef/UniProt protein sequence database where sequences are reduced to one representative from clusters of 50% sequence similarity. Gene Ontology (GO) terms were assigned to each contig with significant UniRef50 hits by using the annotation software Blast2GO [45] with terms taken from GO (SlimGO terms). Pfam scan was performed with the PamScan.pl Perl script provided by Pfam, run with default parameters against Pfam families profiles version 21 downloaded in April 2011 (ftp://ftp.sanger.ac.uk/pub/databases/Pfam/current_release/).

### Predicting resistance gene homologues

The following processes were developed for the prediction of genes involved in plant resistance against pathogens; a lexical search of the GO terms predictions and an HMM approach. The lexical search searches for the terms “nblrr” or “nb-lrr” in the annotation of the GO terms. The HMM approach uses the profile of the NB-ARC domain, obtained from Pfam (version 14), to scan the contig sets by the hmmscan program of the HMMER suite (version 3.0). A strict domain matching e-value of 0.1 was used to select for the NB-ARC hits, as this cutoff filters out most false positives [46] (see also HMMer user guide ftp://selab.janelia.org/pub/software/hmmer3/3.0/Userguide.pdf). Subsequently, within the set of NB-ARC hits a collection of LRR HMM profiles (an intracellular LRR Pfam clan [47]) was scanned (domain e-value 0.1) for the prediction of contigs that have both an NB-ARC and an LRR hit. These were considered members of the NB-LRR protein family. A set of previously predicted NB-LRRs from the four sequenced grass species [38], constituted the grass NB-LRR sequence set and was used as a substrate for the BRBH analysis of the NB-LRR protein family.

### SNP detection

To predict SNPs, 454-reads from each accession individually were aligned back onto the contigs and isotigs from the ‘combined Newbler default’ assembly. The alignment considers the sets of contigs as a pseudo-reference transcriptome and proceeds with the multiple alignment of the reads on the reference sequences. The alignments were performed by Mosaik (http://bioinformatics.bc.edu/marthlab/Mosaik) and the output processed by GigaBayes [48] (http://bioinformatics.bc.edu/marthlab/Software_Release) for SNP prediction using the ‘two organism’ mode and a minimum of 2 reads coverage per allele. Gigabayes predicts SNPs based on a Bayesian framework to assess the probability of a SNP being a real polymorphism and it was set up to look for SNPs between the accessions 1644 and 2232.

### Genotyping

A subset of SNPs identified from the combined Newbler default contigs and isotigs were selected to determine the rate of marker conversion (Table S3). Flanking sequence of SNPs was extracted, aligned to rice to avoid exon-intron boundaries, and used as input in Sequenom Assay Designer v 4.0. Default parameters were used to design several iPLEX pools, with a maximum iPLEX size of 30. Clustering was performed using the default parameters in the software package Typer Analyzer, version 4.0.21.66, Sequenom Inc. Replicated genomic DNA from accessions 1644 and 2232 and 12 F_2_ lines was included in the analysis to determine the potential of converting predicted SNPs into co-dominant markers.

## Supporting Information

Text S1 **List of predicted SNPs.** File containing all SNPs predicted from Gigabayes for the three selected assemblies as well as the subset of SNPs predicted from our high quality SNP prediction approach.
(BZ2)

Figure S1 **Assessment plots for all 13 assemblies.** (A) Contigs (and isotigs where appropriate) scatter-plots and marginal histograms of all 454 de-novo transcriptome assemblies. Scatter-plots (in log-log scale) of the contigs/isotigs length vs. the number of reads that generated each contig/isotig. (B) Table of the self-BLASTn hits for each assembly.
(PDF)

Figure S2 **Boxplots of coverage along the relative length of contigs.** Boxplots of the 454 read coverage per nucleotide position (relative to 100 nucleotides) of all the *Ae* *. sharonensis* assemblies.
(PDF)

Table S1 **Blast2GO organism hits.** A list of the organism names of the first BLAST hit (against nr) from all the three *Ae* *. sharonensis* selected assemblies.
(XLSX)

Table S2 **Non-redundant sequence set characterisation.** The BRBH, UniRef, GOterms, nr hits as well as the predicted SNPs for each of the 62,243 non-redundant sequence set of *Ae* *. sharonensis* .
(XLSB)

Table S3 **SNPs tested by Sequenom genotyping.** Details of the 59 SNPs from the combined Newbler default assembly that were tested for marker conversion by Sequenom genotyping.
(XLSX)
