# Supplementary material for: Characterisation and Analysis of the Aegilops sharonensis Transcriptome, a Wild Relative of Wheat in the Sitopsis Section
Source: PLoS One. 2013 Aug 8;8(8):e72782. doi: 10.1371/journal.pone.0072782 (PMC3738571; doi:10.1371/journal.pone.0072782)
Supplement: Figure S1 — Assessment plots for all 13 assemblies. (A) Contigs (and isotigs where appropriate) scatter-plots and marginal histograms of all 454 de-novo transcriptome assemblies. Scatter-plots (in log-log scale) of the contigs/isotigs length vs. the number of reads that generated each contig/isotig. (B) Table of the self-BLASTn hits for each assembly. (PDF) [file pone.0072782.s002.pdf]

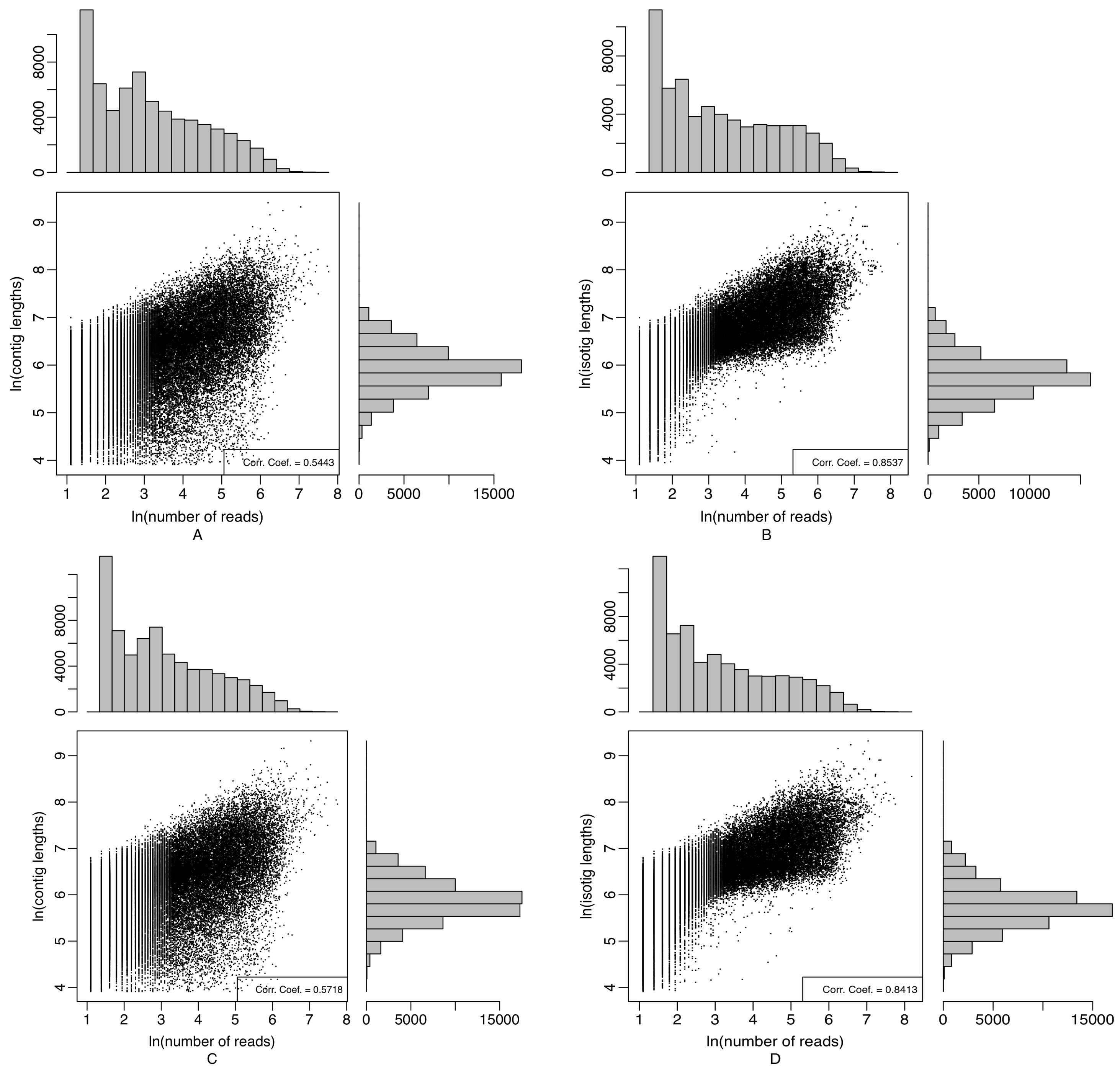

ln(contig lengths)
ln(number of reads)
Corr. Coef. = 0.5443
A
ln(isotig lengths)
ln(number of reads)
Corr. Coef. = 0.8537
B
ln(contig lengths)
ln(number of reads)
Corr. Coef. = 0.5718
C
ln(isotig lengths)
ln(number of reads)
Corr. Coef. = 0.8413
D

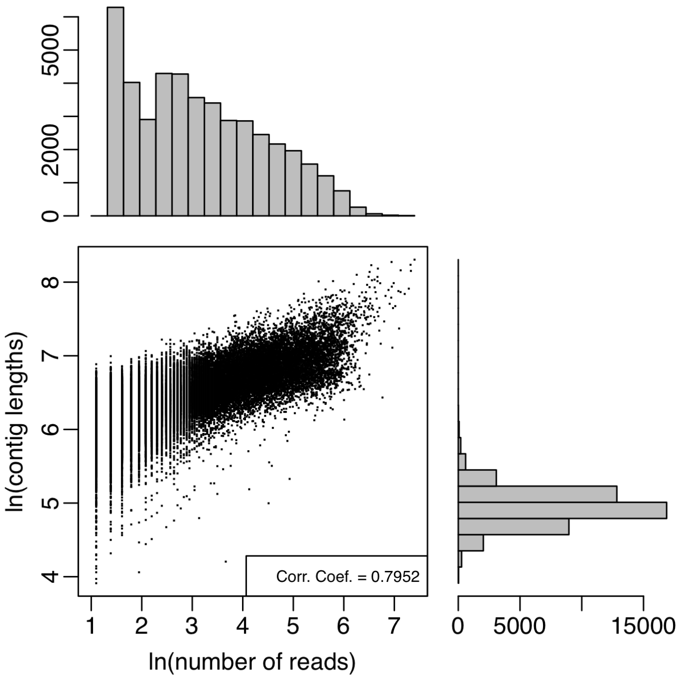

E

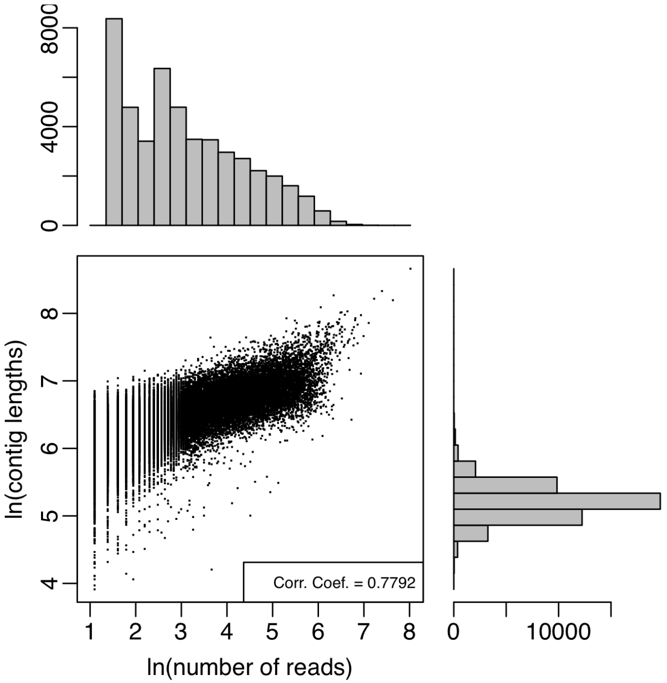

F

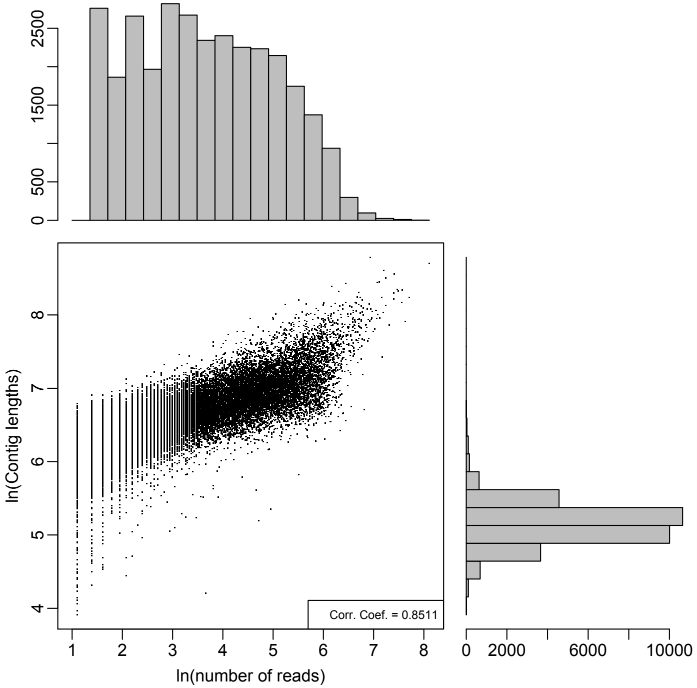

G

Additional File 1a – *Combined Assemblies*. Scatter-plots of the length of contigs over the number of reads that have been used to construct the contig. The histograms in the “X” and “Y” axes represent the distribution of contig lengths and number of reads respectively.

A: Newbler default assembly contigs.
B: Newbler default assembly isotigs.
C: Newbler strict assembly contigs.
D: Newbler strict assembly isotigs.
E: CAP3 default assembly contigs.
F: CAP3 strict assembly contigs.
G: CAP3 relaxed assembly contigs.

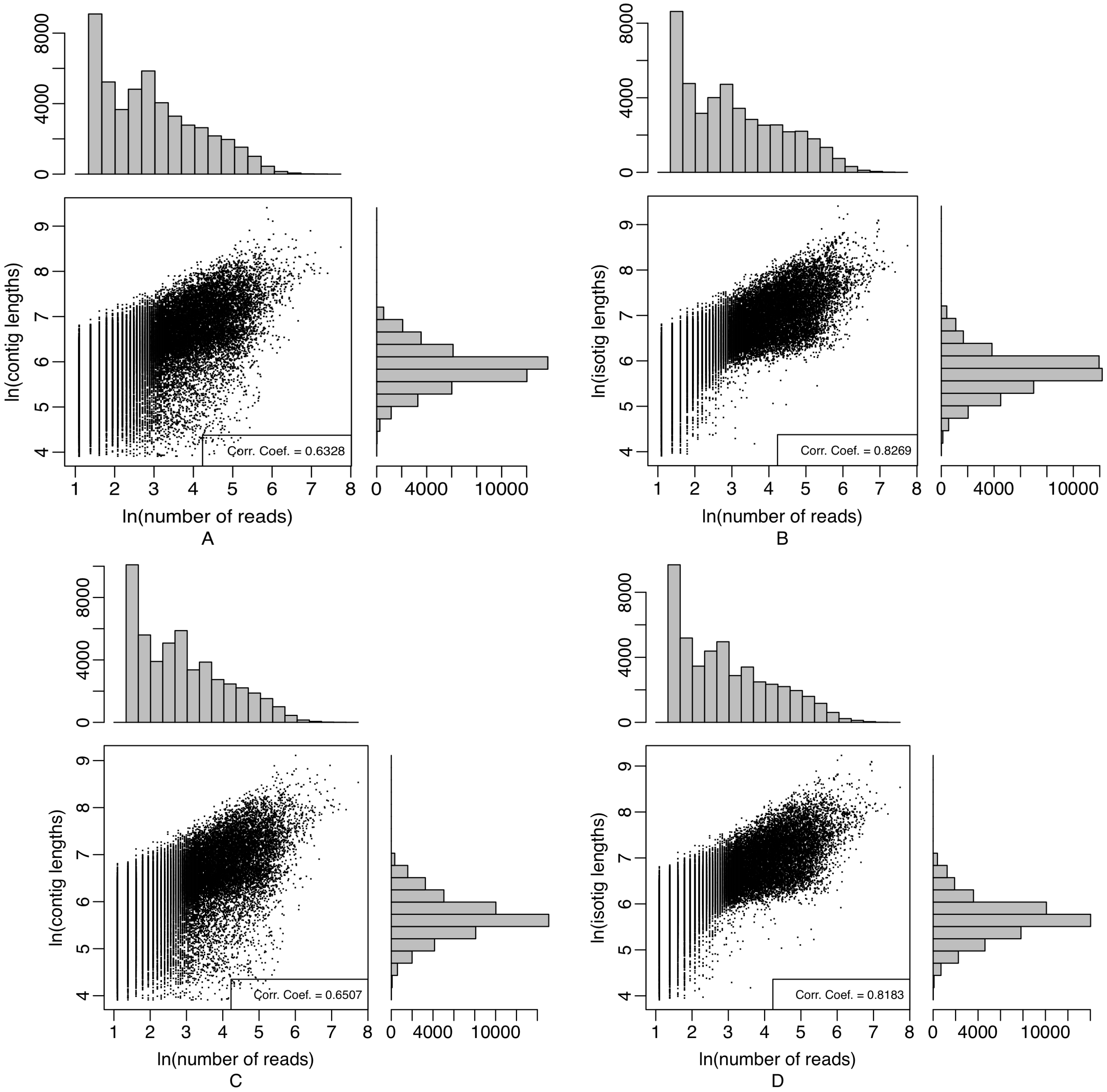

Corr. Coef. = 0.6328
ln(contig lengths)
ln(number of reads)
A
Corr. Coef. = 0.8269
ln(isotig lengths)
ln(number of reads)
B
Corr. Coef. = 0.6507
ln(contig lengths)
ln(number of reads)
C
Corr. Coef. = 0.8183
ln(isotig lengths)
ln(number of reads)
D

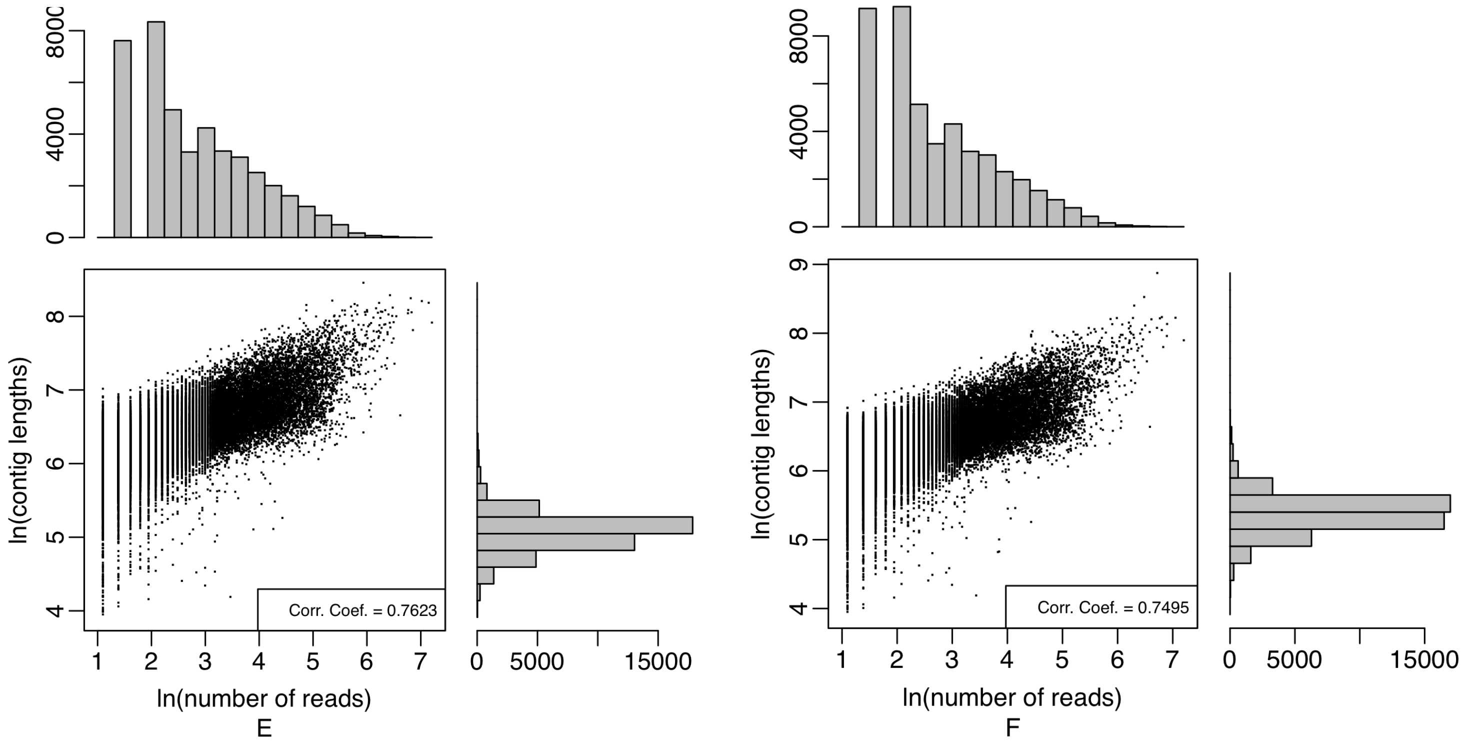

Assemblies of the 1644 Accession.
Scatter-plots of the length of contigs over the number of reads that have been used to construct the contig. The histograms in the "X" and "Y" axes represent the distribution of contig lengths and number of reads respectively.

A: Newbler default assembly contigs.
B: Newbler default assembly isotigs.
C: Newbler strict assembly contigs.
D: Newbler strict assembly isotigs.
E: CAP3 default assembly contigs.
F: CAP3 strict assembly contigs.

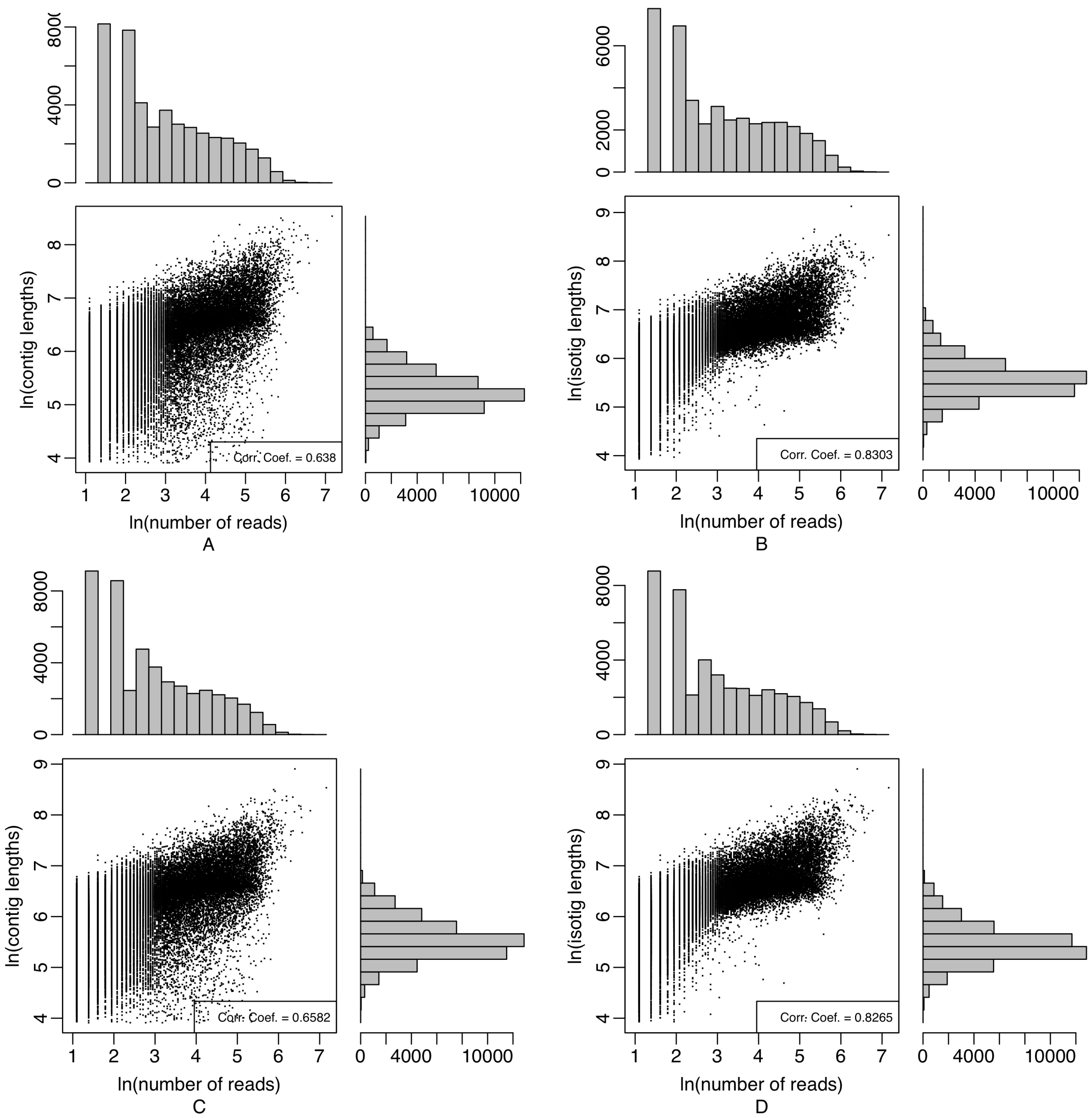

ln(contig lengths)
ln(number of reads)
Corr. Coef. = 0.638
A
ln(isotig lengths)
ln(number of reads)
Corr. Coef. = 0.8303
B
ln(contig lengths)
ln(number of reads)
Corr. Coef. = 0.6582
C
ln(isotig lengths)
ln(number of reads)
Corr. Coef. = 0.8265
D

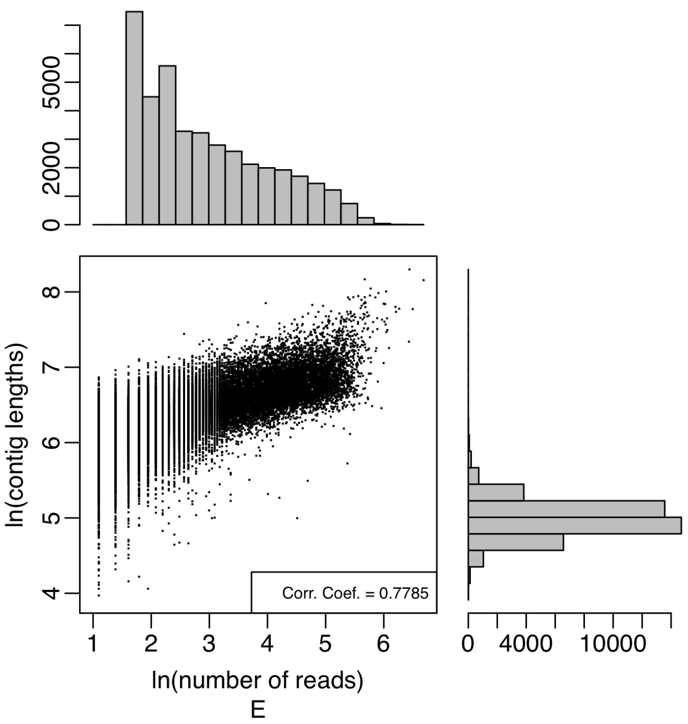

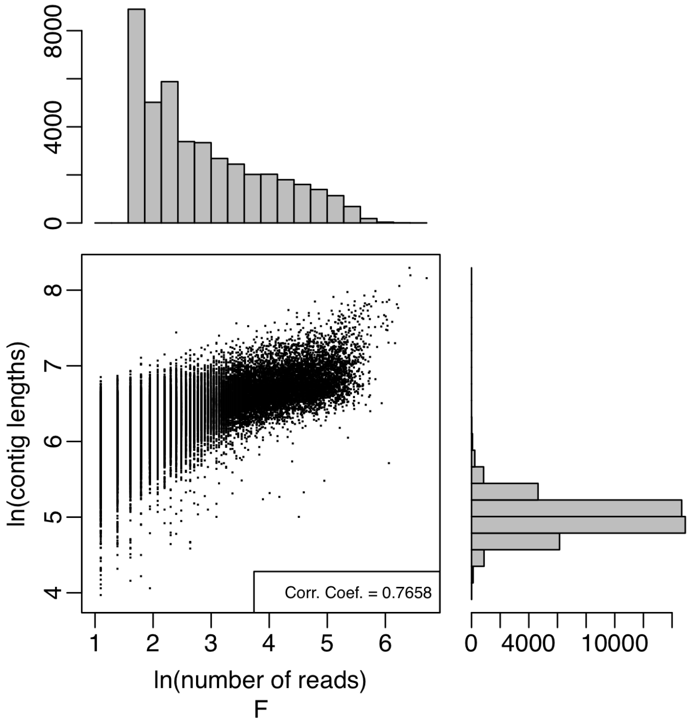

Assemblies of the 2232 Accession.
Scatter-plots of the length of contigs over the number of reads that have been used to construct the contig. The histograms in the "X" and "Y" axes represent the distribution of contig lengths and number of reads respectively.
A: Newbler default assembly contigs.
B: Newbler default assembly isotigs.
C: Newbler strict assembly contigs.
D: Newbler strict assembly isotigs.
E: CAP3 default assembly contigs.
F: CAP3 strict assembly contigs.

Additional file 1b. Self BLASTn hits table.

Assembly	No. sequences	selfBLASTn hits	Proportion
Newbler comb. default Contigs	71,029		
Newbler comb. default Isotigs	62,638		
Newbler comb. strict Contigs	73,109		
Newbler comb. strict Isotigs	63,843		
CAP3 comb. default Contigs	44,961		
CAP3 comb. strict Contigs	48,118		
CAP3 comb. relaxed Contigs	30,609		
Newbler 1644 default Contigs	50,025		
Newbler 1644 default Isotigs	46,013		
Newbler 1644 strict Contigs	50,025		
Newbler 1644 strict Isotigs	47,265		
CAP3 1644 default Contigs	43,900	339	0.7%
CAP3 1644 strict Contigs	45,960	378	0.8%
Newbler 2232 default Contigs	46,619		
Newbler 2232 default Isotigs	42,700		
Newbler 2232 strict Contigs	47,833		
Newbler 2232 strict Isotigs	44,076		
CAP3 2232 default Contigs	40,852		
CAP3 2232 strict Contigs	42,545		
