# Supplementary figures and images for: Characterisation and Analysis of the Aegilops sharonensis Transcriptome, a Wild Relative of Wheat in the Sitopsis Section
Source: PLoS One. 2013 Aug 8;8(8):e72782. doi: 10.1371/journal.pone.0072782 (PMC3738571; doi:10.1371/journal.pone.0072782)

Coverage

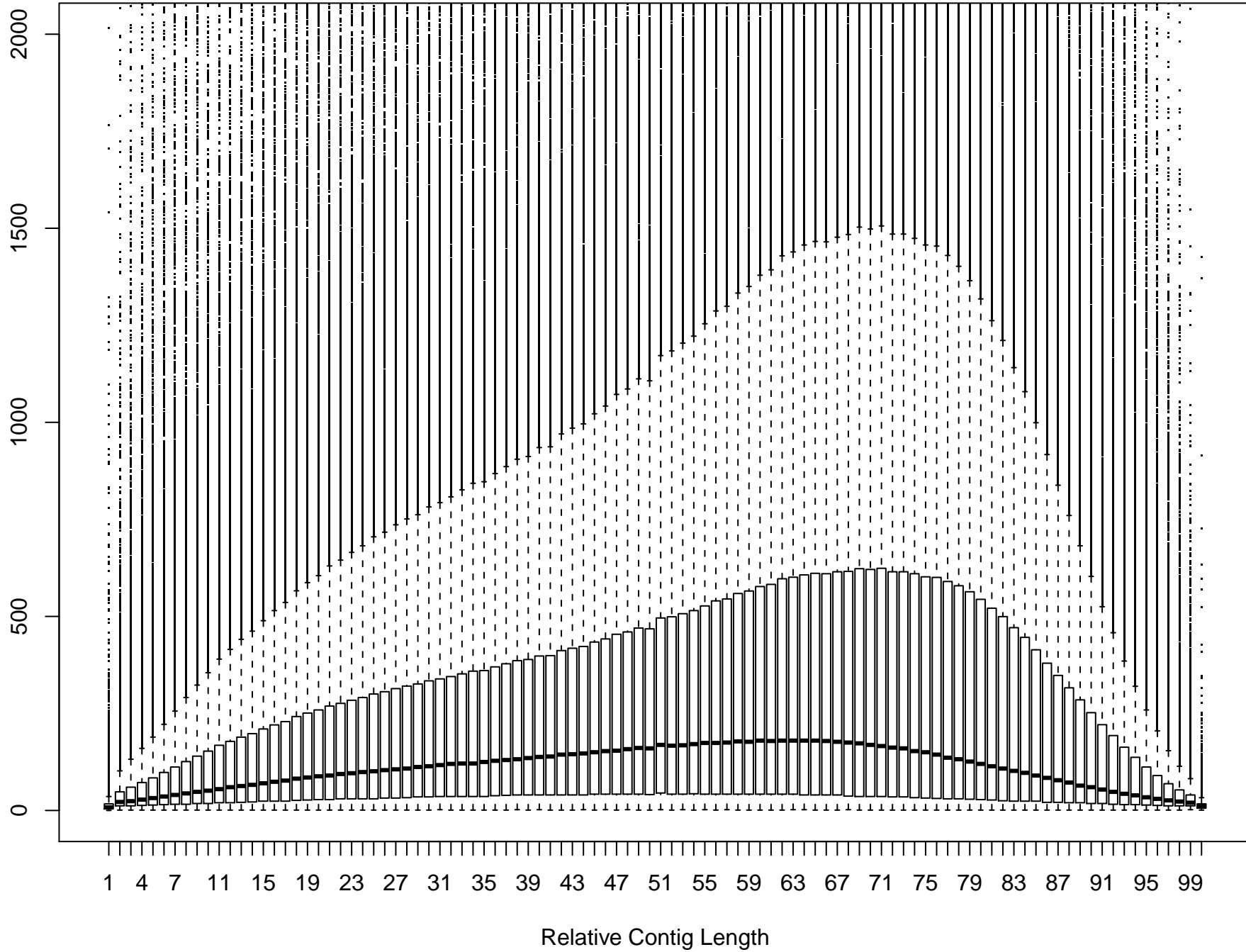

Supplement: Figure S2 — Boxplots of coverage along the relative length of contigs. Boxplots of the 454 read coverage per nucleotide position (relative to 100 nucleotides) of all the Ae . sharonensis assemblies. (PDF) [file pone.0072782.s003.pdf]
